# 1029. Case Study. Interdisciplinary Burn ICU Rehabilitation: Case Study of Self-inflicted Circumferential Neck Burn Recovery

**DOI:** 10.1093/jbcr/irag033.545

**Published:** 2026-04-06

**Authors:** Hadley A Regal, Lily DuRose, Christopher R LaChapelle, Callie M Thompson

**Affiliations:** University of Utah Health Burn Center, Salt Lake City, UT; University of Utah Health, Salt Lake City, UT; University of Utah Health, Salt Lake City, UT; University of Utah Health, Salt Lake City, UT

## Abstract

**Patient Presentation (age range, injury details, relevant history):**

A 25 yo female sustained a self-inflicted, full-thickness circumferential neck burn requiring excision to muscle, biosynthetic dermal substitute (DS) placement, & both sheet & mesh split-thickness skin grafting. Due to the injury's location & severity, she faced high risk for contracture, airway compromise, dysphagia, & significant loss of cervical mobility.

**Clinical Challenges:**

Management of this injury included addressing profound risks to functional outcomes including anterior neck tethering, impaired laryngeal elevation, & long-term range of motion (ROM) deficits. The need for airway protection, preservation of swallowing function, & structural support during DS integration & grafting posed complex rehabilitation challenges within a BICU setting.

**Management Approach:**

Early interdisciplinary care was initiated between Occupational Therapy (OT) & Speech-Language Pathology (SLP). OT applied self-adherent elastic wraps (SAEW) circumferentially to the neck & jaw to manage edema, maintain contour, & support scar remodeling during both the DS & post-graft phases. Application was adjusted daily to protect fragile graft sites & ensure safe healing. SLP conducted swallow assessments postoperatively & implemented a daily orofacial & cervical mobility program aimed at maintaining laryngeal elevation, minimizing anterior tethering, & supporting safe oral intake & communication.

**Outcomes:**

Cervical ROM showed marked improvement over time across all domains (see table in Fig. 1). Swallow function improved steadily, with no signs of aspiration. Therapy teams noted enhanced tissue pliability, preserved neck contour, & no contractures requiring surgical intervention.

**Lessons Learned:**

This case illustrates the power of early interdisciplinary rehabilitation in the ICU. SAEW based compressive support was a simple yet highly effective tool in preserving neck alignment & minimizing edema. Daily SLP involvement supported both structural & functional recovery. Patient engagement was a critical factor in sustained progress.

**Applicability to Practice:**

Burn rehabilitation must begin early & involve coordinated efforts across disciplines. This case supports using practical tools like SAEW & daily mobility protocols to prevent long-term morbidity. Interdisciplinary approaches can yield high-functioning outcomes without reliance on traditional inpatient rehab pathways.

